# Maxwell's Daemon: Information versus Particle Statistics

**DOI:** 10.1038/srep06995

**Published:** 2014-11-11

**Authors:** Martin Plesch, Oscar Dahlsten, John Goold, Vlatko Vedral

**Affiliations:** 1 Institute of Physics, Slovak Academy of Sciences, Bratislava, Slovakia; 2 Faculty of Informatics, Masaryk University, Brno, Czech Republic; 3 Department of Physics, University of Oxford, Clarendon Laboratory, Oxford, OX1 3PU, UK; 4 The Abdus Salam International Centre for Theoretical Physics, 34014 Trieste, Italy; 5 Center for Quantum Technology, National University of Singapore, Singapore

## Abstract

Maxwell's daemon is a popular personification of a principle connecting information gain and extractable work in thermodynamics. A Szilard Engine is a particular hypothetical realization of Maxwell's daemon, which is able to extract work from a single thermal reservoir by measuring the position of particle(s) within the system. Here we investigate the role of particle statistics in the whole process; namely, how the extractable work changes if instead of classical particles fermions or bosons are used as the working medium. We give a unifying argument for the optimal work in the different cases: the extractable work is determined solely by the information gain of the initial measurement, as measured by the mutual information, regardless of the number and type of particles which constitute the working substance.

The laws of thermodynamics are well known for their robustness, namely the fact that they survived both physics revolutions of the 20th century. Neither quantum physics nor relativity have modified our confidence in the fact that the overall energy of a closed system should be conserved, while its entropy tends to the maximum possible value with time. However, quantum systems at low temperatures obey completely different statistics to classical ones. For example bosons tend to bunch in the same area, so one might expect to be easier to establish pressure differences, from which one can extract work. One could thus even imagine that bosonic and/or fermionic statistics might allow us to obtain more work in the same situation than from indistinguishable particles and thus break the second law.

In this paper we show that the performance of a work cycle with indistinguishable particles depends on the information one has and is capable of obtaining about them. The type of particles doing the work, be they classical or quantum, distinguishable or indistinguishable, is only of secondary importance. Our work clarifies why the basics of thermodynamics are independent of particle statistics, a fact that makes thermodynamical laws all the more remarkable. Furthermore, it emphasises that the second law ought to most appropriately be phrased in terms of a trade-off between information gained and work done and holds for all types of particles.

Maxwell's daemon systems make use of extra knowledge beyond that of a thermal observer to extract work. Investigating such systems has helped to clarify and develop statistical mechanics. A particularly clean and widely considered type of Maxwell's daemons are called Szilard engines, often based around a cylinder with one (or more) particles inside it^1^,^2^. The engine works by dividing the cylinder into two halves by an impenetrable barrier and measuring the position of the gas particle. Following the measurement, work can be extracted from this device by exploiting the pressure created by the particle on the barrier. The optimal amount of work is given as *kT* ln(2) with *k* being the Boltzmann constant. Bennett famously showed that the Szilard engine daemon shifts entropy into a memory, is thus not a closed cycle, and if one tries to make it one by resetting the memory, the work cost for this cancels out the initial work gain^3^.

The quantum versions of such engines have also been investigated, a notably difference being that the insertion and removal of the barrier in the middle costs/gives work^4^,^5^,^6^. Interesting new phenomena occur if one includes more particles into the SZE. In e.g.^3^,^6^,^7^ it was shown that correlations have a positive impact on the amount of work that can be extracted from the engine, quantum correlations having a particularly strong influence. A closely related type of multi-particle effect is of course particle statistics associated with identical particles. Kim *et.al.*^8^ performed analysis of a SZE with more than one particle, bringing the role of particle statistics into focus. This work was further discussed in^9^,^10^. In^11^ a SZE utilizing purely quantum information was researched.

In this paper we clarify in detail the role of particle statistics in a quantum SZE by placing a particular emphasis on the initial measurement. We consider primarily the low temperature limit as we are interested in quantum effects and these are most strongly manifested in this regime; for finite temperature, indistinguishability of particles is gradually lost due to the emerging distinguishability by energy levels they occupy. We show that the extractable work is directly connected with the entropy extracted from the system by *W* = −*kT*Δ*S* by the measurement performed on the system. The equation can also be rewritten to *W* = *kT*Δ*I*, where Δ*I* is the mutual information established by the measurement. In other words, we find that the extractable work is determined by the information gained during the initial measurement, regardless of whether the working medium consists of distinguishable, bosonic or fermionic particles. By performing a detailed analysis we show that the work costs of the different individual steps within a working cycle do depend on the working medium but conspire to remove this dependence when combined, saving also the validity of the second law.

We proceed as follows. In the first sections we define the SZE with quantum particles forming the working medium and derive the extractable work for full measurements. Then we focus on more general case of partial measurements (where not all possible information about the system is extracted) and connect the information gain of these measurements with extractable work from the system. We finalize with the discussion on possible effects of degeneracies and finite temperature.

## Results

### Szilard engine

Consider *N* quantum particles (bosons, fermions or distinguishable particles) placed in a cylinder kept at a constant temperature *T* via an ongoing interaction between the particles and the walls of the cylinder. This interaction is considered to be fast in the sense that the time scale of thermalization is much smaller than any other time scale used. In the first part of the paper we will also assume the energy levels of the particles in the container, as well as in its parts after inserting the barrier (piston), to be non-degenerate; possible degeneracies will be discussed later on. However this does not exclude the energy levels on the respective sides of the piston having the same energies.

**Figure 1:**
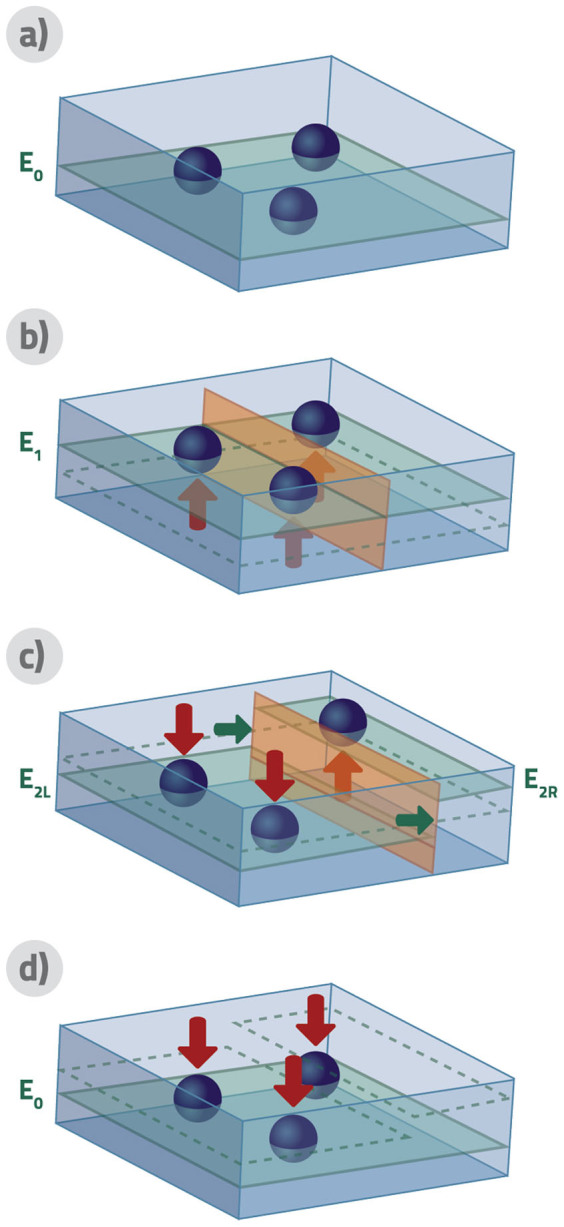
Three-particle Szilard engine. In this figure we represent one working cycle of the Szilard engine with three indistinguishable particles. This scheme holds also for distinguishable particles, when individual parametrization of them is not taken into account in measurement. We start with a container with three particles in the ground state a). After insertion of the barrier, which costs some work associated with the change of energy levels, we perform a measurement on the number of particles on the left-hand side and eventually find two particles there b). In the third stage we move the barrier to its stationary position, changing the energy levels and extracting some work c). In the last stage we remove the barrier to extract rest of the work *W*_2_ from decreasing the energy levels d). In total, *W*_1_ + *W*_2_ = *kT* ln(4).

The original SZE with more particles, with measurement not distinguishing particles even if they are distinguishable in principle, works via the following steps, see Fig. 1:

- 1. A piston (modelled by a sufficiently narrow and high potential barrier) is inserted into the container to separate it into two disconnected regions, preventing any tunnelling within the relevant time-scales.
- 2. A measurement is performed to obtain the number of particles on one side of the cylinder. This measurement is complete for indistinguishable particles, but only partial for distinguishable particles. The state of particles in the cylinder, after the wall was inserted and before the measurement is performed, is a mixture of possible states obtained via the measurement rather then their coherent superposition. This is due to the fact that interactions with the walls of the cylinder will not only fix the temperature of the gas during the process, but also perform a measurement in the number basis in each part of the cylinder, once the barrier is introduced into the cylinder.
- 3. Depending on the result of the measurement the piston will be allowed to move quasi-statically to a position where the side-ways force acting on the piston will be zero. Exact conditions to reach this position shall be discussed later.
- 4. The piston is then quasi-statically removed by decreasing the strength of the potential.

In the *classical* case there is no need to invest work into the engine except during the erasure procedure - insertion of the piston, its removal as well as the measurement are considered to be “for free”. The only stage of extraction of energy is the movement of the piston resulting from the unequal pressure on either side. In contrast, in the the *quantum* case one inevitably has to invest energy to create the barrier, as all energy levels of the particles are increased due to decrease of the available volume. This work can however be recovered during the movement and removal of the barrier (steps 3 and 4). In this picture the stages of movement and removal of the barrier need not anymore be considered as independent actions. To show this, let us define a general force in the form *F* = *∂_λ_E* with *λ* being a general parameter and *E* the total energy of the system. In the third step *λ* can represent the position of the barrier and *F* will be the standard force. In the fourth step (only relevant in cases when not all particles have been found on one side of the barrier) *λ* will be a general parameter associated with the height of the barrier (e.g. strength of the potential forming the barrier) and *F* the respective force. In both cases, the forces can be utilized to extract work by changing the parameter *λ* in a reversible way.

Therefore, throughout the manuscript we will only work with three phases of the engine - insertion, measurement and movement/removal phase. We will denote work gained (or invested, with negative sign) during the insertion of the piston *W*_1_ and the combined work-gain of steps 3 and 4 shall be called *W*_2_. Four stages described above represent a closed cycle up to the fact that the result of the measurement in step 2 is still stored somewhere. For a full restoration of the original setting one has to erase this information^3^.

Within most of this paper we will focus on the low temperature limit, where all particles occupy the lowest possible energy level. Here, the quantum effects of the engine are expected to manifest themselves in the strongest way - for high temperatures all particles became essentially distinguishable as each can be labelled by its energy. In what follows we shall denote the energy levels of the particles in the cylinder without the barrier as *E_i_*. We imply the condition 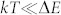, where Δ*E* represents the difference between any two energy levels that could be potentially occupied - for bosons and distinguishable particles it is just the difference between two lowest energy levels of the system *E*_2_ − *E*_1_ and for fermions Δ*E* represents the difference between the Fermi energy and the nearest higher energy level. In the first part of this paper we will also consider the system to be non-degenerate, effect of degeneracies will be discussed later. Under these assumptions the partition function of any system consisting of *N* bosons or distinguishable particles will be *Z_b_* = exp (−*NE*_1_/*kT*) with *E*_1_ being the lowest energy of the system. For fermions in a non-degenerate system the partition function will have the form 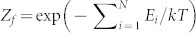.

### Work extraction in different steps

We will now calculate the work that can be extracted from the closed cycle of the SZE. We will use the fact that the work one can extract going between two thermal states with partition functions *Z_A_* for initial state and *Z_B_* for final state is given by 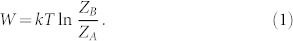
The legitimacy of using this formula even for quantum particles is discussed in the Methods section.

#### Insertion of the barrier

Let us denote the energy levels on the left (right) of the barrier after its insertion as 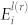. For *bosons* there are two distinct possibilities for this step. The first one corresponds to the case where we do not insert the barrier in the middle of the system; without the loss of generality we can define 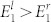. Quantities for this case shall be labelled by index *n* for non-degenerate and the final partition function will be 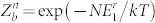, which physically means that all particles condensed in the lower potential well. This will result in a final work 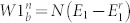. Irrespective of the exact form of the potential defining the cylinder one can expect 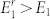, as the “living space” of the particles has decreased, and thus the work performed is negative.

The second possibility for *bosons* corresponds to the case when the barrier is introduced in the middle of the system, i.e. 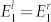 with precision 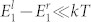. Under such an assumption the partition sum will be 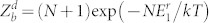 and the work 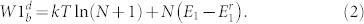
For such a degenerate case all relevant quantities shall be indexed by *d*.

For *distinguishable particles* the situation is quite similar to the above bosonic case. Again there is no limit on the number of particles occupying the lowest energy level. The only difference is for the degenerate case, where the particles can choose their positions without change of the total energy of the system. Here the number of possible configurations will be 2*^N^* and the partition function will be 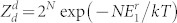. Hence the extractable work will be 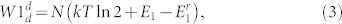
corresponding to the case of simply joining *N* independent SZEs.

For *fermions* the situation is distinctly different. We define *j* as the level for which following inequality holds: 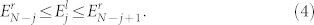
First let us examine the case where there is a sharp inequality on the right hand side of (4). The final partition function will be 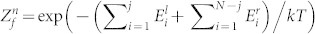 and the extractable work will be 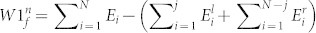. In contrast, for equality on the right hand side of (4) we get a degeneracy in the energy levels of the whole system when the fermion at the Fermi level can freely choose either side of the cylinder. The partition function then reads 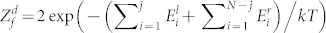 and the extractable work will be 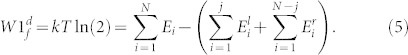
It is notable that in contrast to the bosonic case there are potentially many (in the order of *N*) possibilities for choosing the position of the barrier to reach the equalized position, but insertion into the middle of the container will lead to non/zero work extraction only for *N* odd.

#### Measurement

In the second step a measurement on the system is performed. This is only non-trivial if the energy levels of the system (after inserting the barrier) are degenerate (with partition functions and works labelled by *d*) as in the other case it is just a single-outcome measurement confirming that all bosons/distinguishable particles are in the deeper well (larger part of the container) or the fermions are distributed within the container in a way expectable by the distribution of energy levels.

In the non-trivial bosonic case, the full measurement will have *N* + 1 possible outcomes counting the number of particles on the left hand side. In the fermionic case the measurement will be binary and for distinguishable particles the number of possible outcomes will be 2*^N^* (specifying the left - right position of every single particle).

#### Movement and removal of barrier

In the *classical* case one would extract the work by simply moving the barrier to its equilibrium position. All extractable work would be extracted within this phase for an infinitely narrow barrier. On the other hand, if the barrier was removed from a different position than the equilibrium one, part of the potential work would just dissipate due to mixing of gases with different pressures.

In the *quantum* case the situation is much more subtle. Here the extraction of work is not straightforward to define physically in connection with possible storages of energy. However, if we stick to the standard definition of the generalized force as 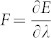 with *λ* being a parameter of the barrier (e.g. its position during the movement phase or height during the removal phase) and accept that any such force can be utilized to perform work, we can calculate the extractable work from the partition functions without making specific assumptions on the process itself.

For both bosons and distinguishable particles the initial partition sum of the system is 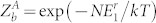 and final is *Z_b_*. Therefore we get as the extractable work 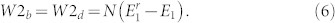
For fermions the partition sum is 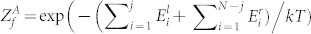 and the final one *Z_f_*; the resulting work is 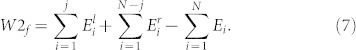


### Total work

The total work gained (or consumed) during the relevant parts of the cycle is given by the sum of the work consumed for inserting the barrier *W*_1_ and the work extracted by the movement and removal of the barrier *W*_2_. For non degenerate cases, for all different kinds of particles this work is *W_b_* = *W_d_* = *W_f_* = 0. This is due to the simple fact that in the low-temperature limit the particles would always choose the lowest energy level available for them. So unless there is a degeneracy of the levels after inserting the barrier, the measurement is trivial and its result can be predicted with certainty before it is actually performed. Such a measurement would not remove any entropy from the system and thus cannot lead to work extraction.

The degenerate cases are much more interesting. In the case of distinguishable particles *W_d_* = *NkT* ln(2), for bosons we get *W_b_* = *kT* ln(*N* + 1) and for fermions *W_f_* = *kT* ln(2). All these results (even for non-degenerate cases) are connected by a unifying formula of the form 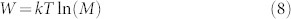
with *M* being the number of possible measurement outcomes, each of them occurring with equal probability. The work corresponds to the energy needed to erase a memory able to store the result of the measurement.

**Figure 2:**
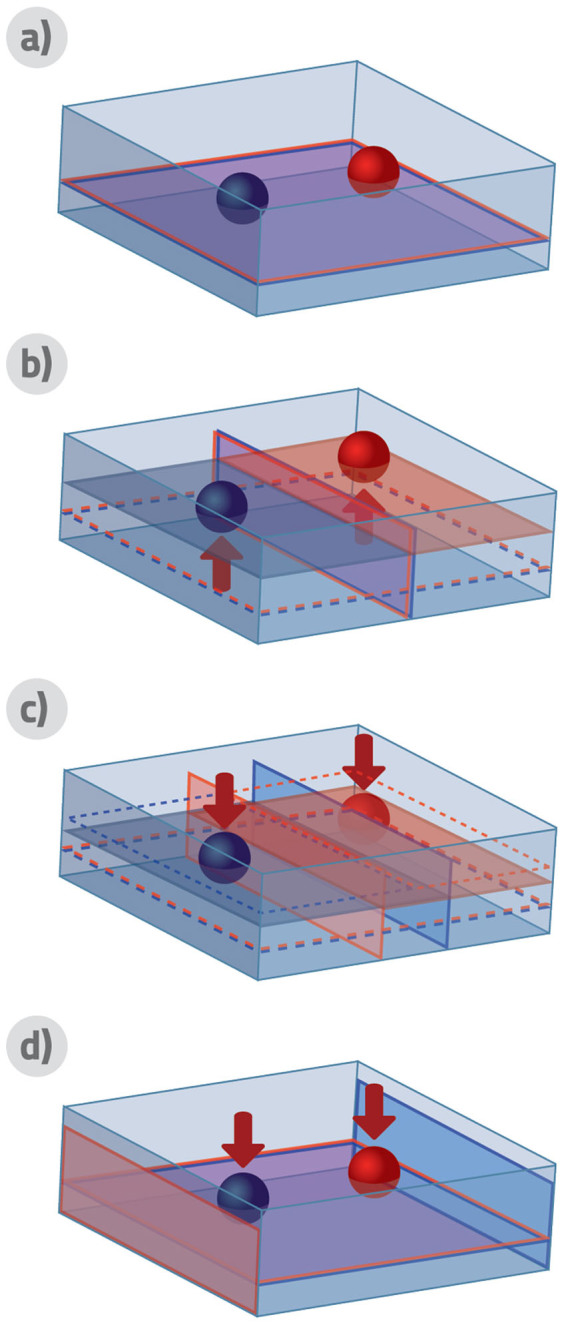
Work extraction for two distinguishable particles. In this figure we represent how energy is extracted from a Szilard engine containing two distinguishable particles. We start with a container with both particles in the ground state a). Filters for both particles are inserted into the middle of the container and the measurement eventually reveals the “blue” particle on the left hand side and the “red” particle on the right hand side b). In the third stage filters are gradually moved to respective sides to utilize the pressure force by individual particles c). In the last stage d) filters are on the edge of the container and can be removed without any work gain from the sides of the container. In total, *W* = 2*kT* ln(2).

Let us briefly discuss here the consequences of this result for distinguishable particles. Importantly, the formula for extractable work (8) not only holds for the average extractable work, but it is also valid as a single-shot formula that guarantees the work to be extracted for every possible measurement outcome. This in particular holds for the insertion of the barrier in the centre of the container for distinguishable particles and finding the same number of particles on each side. This counter-intuitive fact is possible by utilizing filters - semipermeable membranes that would allow to penetrate all particles but a specific one. Possible existence of this kind of filters is guaranteed by the distinguishability of the particles itself: If there is a way how to distinguish individual particles by a measurement, this measurement can be utilized to construct the specific filter. Now the membrane inserted into the center of the container will consist of a set of *N* filters, one for each particle. Each of these filters would then be moved towards the respective side of the container, gaining *kT* ln(2) work, together corresponding to *N* independent SZEs. For an example for two particles, see Fig. 2.

### Partial measurements and information–work relation

One may consider another way of exploiting particle statistics to try to violate the second law, that is by doing a coarse-grained measurement. Let us define a coarse-grained measurement with *M* outcomes labelled by *m* (running from 1 to *M*), each occurring with probability *p_m_*. Such a measurement can be designed from a more general measurement with *N* > *M* outcomes and joining these outcomes into groups indistinguishable for the coarse grained measurement *M*. This coarse-graining might have different reasons, from not being able to measure the exact number of particles on each side of the cylinder to not being able to individually recognize the distinguishable particles. We will show here that irrespective on the reason of the incompleteness of the measurement, the extractable work is given solely by the information gain of the measurement given by the probability distribution of the measurement results.

First note that as *W*_1_ does not depend on the measurement, it will not change if a coarse-grained measurement is used instead of a complete one.

For bosons and distinguishable particles, the partition sum after performing the measurement and obtaining the outcome *m* will have the form 
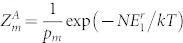
. Further, after the removal phase the partition sum will change to 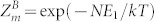. The extractable work for a specific outcome *m* will thus be 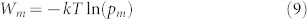
and the average extractable work will be 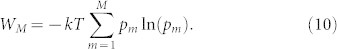
For fermions, the total measurement is always a binary-outcome measurement, therefore no coarse-grained measurements can be defined. However, the above Eq. (10) still holds by taking *M* = 2 and *p*_1_ = *p*_2_ = 1/2.

From (10) we see that the extractable work does not in fact depend on the actual number and type of particles in the system, but only on the measurement and its possible outcomes (taking into account that possible measurements are limited by the number and type of particles in the system). Given a fixed number of possible outcomes *M*, the extractable work is maximized for a measurement with equal probability of each outcome and will gain *W*_max_ = *kT* ln(*M*). We see that the maximal extractable work is directly associated with the measurement with the highest information gain, given the number of possible outcomes. We further discuss the optimality of such a measurement in the Methods section.

Note here that unlike for full measurements, in case of coarse-grained measurements the extractable work might be higher for specific results comparing to the average. Yielding a highly improbable outcome with a very small *p_m_* the extractable work *W_m_* might be much higher that *W_M_*. But still *W_m_* will be upper bounded by *kT* ln (*N*) with *N* being the governing full measurement from which the coarse-grained measurement was induced.

We note also that the result in Eq. (10) can we rewritten as *W* = *TS* with 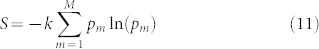
being the entropy of the measurement outcomes. This definition exactly corresponds to the definition of Gibbs entropy except that here one deals with the probabilities of measurement outcomes rather than with the probabilities of microstates. One might view this correspondence in the following way: by removing entropy *S* from the system by a measurement one is able to extract exactly *W* = *TS* of work from the system before it turns back to its original state. The extracted entropy has to be stored somewhere, or erased, costing exactly the same amount of work again^3^.

Eq. (11) can further be rewritten in terms of mutual information. Let *p_s_*(*n*) be the probability distribution of the states of the system (thus 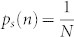 for all *n*). Let *p_m_*(*m*) be the probability distribution on the measurement apparatus with *M* possible outcomes. The joint probability distribution *p_ms_*(*m*, *n*) will be non-zero only if system state *n* will lead to measurement result *m* and in this case will read 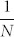. It is easy to see that the resulting mutual information between the probability distributions of the system and the measurement apparatus will be 
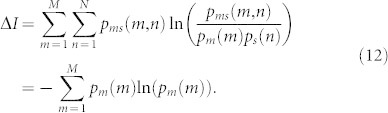
Thus one can write *W* = *kT*Δ*I*.

#### Measurement ignoring distinguishability of particles

Let us briefly discuss here a specific example of a coarse-grained measurement, namely the measurement of number of particles on the left-hand side of the barrier for distinguishable particles. Such a measurement is easily experimentally realizable, as the number of particles on a side of the piston is more easily measurable comparing to a measurement that would individually recognize each particle.

Intuitively one expects the probability of different outcomes to be concentrated around the balanced state, with approximately equal number of particles on each side. Moreover, these outcomes are expected to yield only little work. This intuition is confirmed by exact calculation which yields the expression for the average extractable work 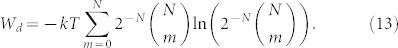
This is for all *N* lower than *W_b_* for *N* bosons, where this kind of measurement represents a full measurement of the system. With growing *N* the extractable work *W_d_* drops down to 
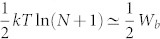
12. This can be explained by the smaller information value of the measurement outcome (with the same number of possible outcomes *N* + 1) for distinguishable particles in comparison to bosons - whereas for bosons any result is equally probable, for distinguishable particles only the results close to balanced are more likely.

In the very unlikely event of finding all particles on one side of the piston we will be able to extract a much higher amount of work than (13), namely *NkT* ln(2), what is exactly the amount corresponding to the full measurement on *N* distinguishable particles. This is due to the fact that a measurement result obtaining all particles on one side is not coarse-grained at all. On the other hand for the most probable outcome with balanced number of particles on each side the extractable work will be 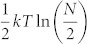, thus only slightly lower than the average. Detailed calculations are available in the Methods section.

### Degeneracy

Let us briefly discuss the possible degeneracies of energy levels in the system. For bosons and distinguishable particles they only play a role for the lowest energy level. If the degeneracy can be revealed by the measurement, the system will correspond to an advanced SZE with more than one barrier and more complicated measurement. If the degeneracy cannot be revealed by the measurement, the measurement has to be considered as coarse-grained. However, as the probabilities for respective outcomes will be still equal (each coarse-grained measurement outcome will join the same number of full-measurement outcomes) and thus having full entropy, the resulting work will not change at all.

For fermions, if the Fermi energy level of the system is degenerate, more than one fermion can occupy the same energy. This case represents a kind of fermionic – bosonic combination that increases the number of particles actually performing work in the system from one to the degeneracy degree of the Fermi level. In the marginal scenario of the degeneracy reaching *N*, fermions behave exactly like bosons, condensing into the lowest energy level and performing the same amount of work as bosons (if the degeneracies cannot be revealed by measurement) or distinguishable particles (if degeneracies can be revealed by the measurement).

### Effects of finite temperature

If the condition 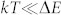 for low temperature limit is not fulfilled, the situation becomes more complicated. Partition sums will include also terms with higher energy levels, as the probability of finding one of the particles in these levels will not be negligible anymore. The consequences can be summarized under two main topics.

First, indistinguishable particles will become partly distinguishable due to their different energy. Bosons can be viewed as divided into distinct groups of indistinguishable particles labelled by their energy; the which-side measurement, complete for cold bosons, becomes then coarse-grained for warm ones. Information value of this measurement will gradually decrease with increasing temperature up to the point, where each boson could be potentially distinguishable by its energy, leading to a decrease of extracted work.

For fermions, effects of finite temperature will be similar to degeneracy effects - one will be able to utilize some more complicated than binary measurement, as fermions might occupy also other than Fermi energies, leading to the increase of the extractable work.

The second effect is connected with a richer structure of possible extraction procedures. For cold bosons and distinguishable particles, the barrier could be inserted only in the middle of the cylinder to extract work, for fermions only in a number of distinct places. For warmer particles the outcomes of the measurements even for non-symmetrical insertion of the barrier will not be trivial anymore, hence work could be extracted also in these cases.

In the high temperature limit, differences between different particle statistics will be completely eliminated and they will all behave similarly to a classical gas, where work can be extracted by insertion of the barrier in any point into the cylinder, and the work costs for insertion of the barrier are negligible compared to work extracted.

## Discussion

We performed a detailed analysis of a specific realization of a Maxwells daemon – Szilard engine, where the working medium consist of either distinguishable particles, bosons or fermions. We showed that the extractable work is determined by the information gain of the measurement performed on the system, regardless of the working medium. We demonstrated in detail how things conspire to remove the dependence on the working medium in that sense. This latter contribution is arguably of the same type as Bennett's exorcism of Maxwell's daemon^3^ in that it shows in what exact way the second law is not violated in this process.

We also showed that if a full measurement on the system is performed (which is associated with a different amount of information gain in different cases), distinguishable particles exhibit a much larger potential to deliver work (scaling linearly with the number of particles) relative to bosons (where it scales logarithmically). Fermions can only provide a fixed amount of work independent of the number of particles in non-degenerate case.

Moreover we showed that for coarse-grained measurements the extractable work is again determined solely by the information gain of the measurement. This clarifies why the same kind of measurement (measuring the number of particles on either side of the piston) extracts more energy for bosons than for distinguishable particles.

It would be interesting to provide experimental evidence for the results obtained, especially for the possibility to extract work with balanced measurement outcomes. One could think about cold atoms kept at a stable temperature by a (larger amount) of different atoms, as suggested in^8^. Another option would be to use photons in micro-cavities as the working media. Here the confinement potential is easily controllable and the barrier could be realized simply as an inserted mirror.

## Methods

### Legitimacy of using 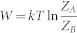 formula for quantum particles

We show here that the above-mentioned formula can indeed be used even in cases where the working medium consist of quantum particles. First define *F* to be a general force with *F* = *∂_λ_E*, where *E* is the energy of the system the force is acting on, and *λ* be the parameter varied when the work is done. One may for example take *E* to be the energy of a state in the Szilard engine, and *λ* the parameter that controls the position of the barrier. Then if that state is occupied and *E* depends on *λ* it will cost energy to do this. The claim now is that 〈*F*〉 = *∂_λ_* (−*kT* ln *Z*), where 
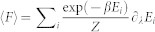
. That the claim holds can be seen from a few lines of calculation. One may worry that this uses the standard Gibb's state and does not hold for bosonic and fermionic statistics. However these are also Gibb's states on the allowed energy levels at the many-particle level. Therefore the same argument carries through also in that case. By considering a quasi-static isothermal sequence of such infinitesimal work extractions with *d*〈*W*〉 : = 〈*F*〉*dλ* one sees that the total *W* is indeed 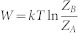. It is also shown in^13^ that 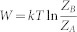 is not only the average work but the maximal work that can be guaranteed to be extracted with probability 1.

### Why information gain determines optimal work

We now show that the protocols introduced above are optimal in that the information gain in the measurement is the best that one can do regardless of the type of particles making up the working medium. We consider three systems: *g* for the working medium (gas), *m* for the memory and *r* for the work reservoir.

Consider firstly the information gain in the measurement. Define this as Δ*I*(*g* : *m*): = Δ(*S*(*ρ_g_*) + *S*(*ρ_m_*) − *S*(*ρ_gm_*)) relative to before and after the measurement. Before the measurement *I*(*g* : *m*) = 0, as the memory is taken to be in some default pure state |0〉*_M_*. The measurement is, optimally, reversible such that *S*(*ρ_gm_*) is the same before and after the measurement. Moreover we demand that *ρ_g_* is unchanged by the measurement as the thermal and decohering reservoir is constantly projecting the system into basis states of the measurement performed. Thus 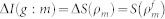 as evaluated after the measurement (*f* denotes final).

We now show that the optimal work that can be extracted is indeed given by Δ*I*(*g* : *m*) = *S*(*ρ_m_*), under certain definitions and conditions. We are interested in the energy change in the work reservoir *r* from between the premeasurement state to the post-extraction state, but without the resetting of the memory having been done. We demand that the entropy of the work reservoir is negligible both before and after the working cycle and define the work extracted as the change in internal energy of the reservoir Δ*U_r_* : = Δ*Tr*(*ρ_r_H_r_*). (If the entropy of *r* was allowed to change we would define the work as the change in free energy of *r* and the argument would still carry through). For simplicity we also take the work reservoir to be in a pure energy eigenstate before and after the extraction, labelled by |0〉*_r_* and |0 + Δ*E*〉*_r_*, such that Δ*U_r_* = Δ*E*. We wish to show that 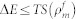. We shall take as our starting point the crucial expression 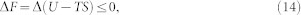
which according to universally accepted models of thermalisation state holds for any system, in equilibrium or not, interacting with a heat bath of temperature *T*, with *U* = *Tr*(*ρH*) and *S* = −*trρ* log *ρ*.

Before evaluating Δ*F* we make the assumption that both at the initial and final times the following four conditions hold: (i) The interaction energies are negligible s.t. *U_gmr_* = *U_g_* + *U_m_* + *U_r_*, (ii) The states are product states s.t. *S* = *S_g_* + *S_m_* + *S_r_*, (note that the work extraction would decouple the memory from *g*) (iii) the Hamiltonian of the memory is proportional to the identity s.t. *U_m_* is state-independent, and -as already mentioned- (iv) *S_r_* = 0. It then follows that 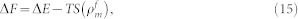
implying that the energy increase in the work reservoir Δ*E* is indeed bounded as 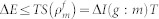.

### Measurement ignoring distinguishability of particles

The probability of finding a specific configuration of *N* distinguishable particles is 2^−*N*^. Having *m* particles on the left hand side is a measurement result coarse-grained in the level of 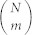, thus having a probability of appearance 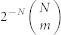. This yields the extractable work for the specific result *m*

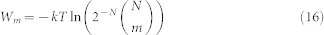
and the average extractable work 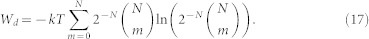
By setting *m* = 0 we get *W*_0_ = *NkT*. On the other hand, by setting *m* = *N*/2 we get approximately 
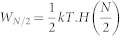
, where *H* stands for Harmonic number. This can be further approximated for large *N* to 
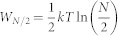
.

## Author Contributions

M.P., O.D., J.G. and V.V. conceived the idea. M.P. and O.D. derived the technical results and together with V.V. prepared the manuscript.
